# Interplay between immune cells and metabolites in epilepsy: insights from a Mendelian randomization analysis

**DOI:** 10.3389/fnagi.2024.1400426

**Published:** 2024-08-07

**Authors:** Kai Wang, Jinwei Yang, Wenhao Xu, Lei Wang, Yu Wang

**Affiliations:** ^1^Department of Neurology, The Third Affiliated Hospital of Anhui Medical University, Hefei, China; ^2^Department of Neurology, The First Affiliated Hospital of Anhui Medical University, Hefei, China; ^3^Department of Neurology, The Affiliated Fuyang People’s Hospital of Anhui Medical University, Fuyang, China

**Keywords:** epilepsy, immune cells, metabolites, causal relationships, Mendelian randomization

## Abstract

**Background:**

Epilepsy is associated with the immune system and metabolism; however, its etiology remains insufficiently understood. Here, we aim to elucidate whether circulating immune cell profiles and metabolites impact the susceptibility to epilepsy.

**Methods:**

We used publicly available genetic data and two-sample Mendelian randomization (MR) analyses to establish causal relationships and mediating effects between 731 immune cells and 1,400 metabolites associated with epilepsy. Sensitivity analyses were conducted to detect heterogeneity and horizontal pleiotropy in the study results.

**Results:**

MR analysis examining the relationship between immune cells, metabolites, and epilepsy revealed significant causal associations with 28 different subtypes of immune cells and 14 metabolites. Besides, the mediation effects analysis revealed that eight metabolites mediated the effects of six types of immune cells on epilepsy and that 3-hydroxyoctanoylcarnitine (2) levels exhibited the highest mediating effect, mediating 15.3% (95%CI, −0.008, −30.6%, *p* = 0.049) of the effect of DN (CD4−CD8−) AC on epilepsy. 1-(1-enyl-stearoyl)-2-linoleoyl-GPE (p-18:0/18:2) levels (95%CI, 0.668, 10.6%, *p* = 0.026) and X-12544 levels (95%CI, −15.1, −0.856%, *p* = 0.028) contributed 5.63 and 8%, respectively, to the causal effect of FSC-A on myeloid DC on epilepsy.

**Conclusion:**

This study revealed a significant causal link between immune cells, metabolites, and epilepsy. It remarkably enhances our understanding of the interplay between immune responses, metabolites, and epilepsy risk, providing insights into the development of therapeutic strategies from both immune and metabolic perspectives.

## Introduction

Epilepsy is a complex and chronic neurological disorder. Epileptic seizures occur when an area of the brain or the entire brain experiences abnormal synchronous discharge of neurons due to factors such as infection or metabolic disruption (Falco-Walter, 2020). It is pathologically characterized by sudden and abnormal brain discharges, which can lead to temporary brain dysfunction (Fisher et al., 2005). The estimated incidence rate of epilepsy is 50 per 100,000 people, affecting over 70 million people worldwide (Thijs et al., 2019). The Global Burden of Epilepsy Report estimated over 125,000 epilepsy-related deaths in 2016, 81% of which occurred in countries with a low or medium sociodemographic index (GBD 2016 Epilepsy Collaborators, 2019). Despite antiepileptic drugs being the main treatment, approximately 30% of epilepsy patients are resistant to drug therapy and rely on alternative interventions such as surgery (Laxer et al., 2014). Epilepsy not only significantly impacts the quality of life of patients but also increases their financial burden through treatment costs.

The development and progression of epilepsy involve complex biochemical processes. For a long time, researchers considered the brain as an immune-privileged area due to the presence of the blood–brain barrier (BBB). However, evidence indicates that both innate and acquired immunity can be rapidly induced within the central nervous system to respond to tissue damage caused by various reasons (Mukhtar, 2020). Moreover, increasingly more evidence shows the involvement of brain vascular dysfunction in the pathogenesis of epilepsy (Marchi et al., 2016; Vezzani et al., 2019). After the BBB is compromised, the infiltration of neural cells, brain microvascular endothelial cells, and peripheral immune cells into the brain parenchyma emerges as an important factor in the occurrence of epileptic seizures (Banks and Erickson, 2010). Immune cells, particularly microglial cells in the brain and T cells in the peripheral blood, are activated during epileptic seizures and release inflammatory factors such as cytokines and chemical mediators (Ravizza et al., 2008; Vezzani et al., 2011; Librizzi et al., 2012). These factors directly damage neurons, promoting the occurrence and persistence of epilepsy (Ravizza et al., 2008). Additionally, the inflammatory response alters the internal environment of the brain, making neurons more susceptible to abnormal discharge (Vezzani et al., 2011).

The association between plasma metabolites and epilepsy is increasingly becoming a focus of research, primarily because these metabolites reveal biochemical changes associated with epileptic seizures. Current studies indicate that more than 200 different metabolic diseases can trigger epileptic seizures, affecting the onset of epilepsy through various pathways (Almannai et al., 2021). For instance, compared to healthy individuals, patients with epilepsy show significant differences in the levels of amino acids, fatty acids, and energy metabolites (Auvin, 2012; Bejarano and Rodríguez-Navarro, 2015). These changes are related to not only alterations in neurotransmitter balance but may also involve inflammatory responses and changes in brain energy utilization and metabolic state (Almannai et al., 2021). Particularly, amino acids, such as the primary excitatory neurotransmitter glutamate and the key inhibitory neurotransmitter gamma-aminobutyric acid (GABA), play crucial roles in the neural transmission processes involved in epileptic seizures. The imbalance between these amino acids in epilepsy patients can cause excessive neuronal excitability, and thereby triggering seizures (Scharfman, 2007; Coulter and Steinhäuser, 2015). Additionally, certain plasma metabolites can interfere with neurotransmission. For example, glycine, acting as a co-agonist for the N-methyl-D-aspartate (NMDA) class of glutamate receptors, plays a crucial role in modulating the excitability of the nervous system. An abnormal increase in the concentration of glycine in the blood may lead to excessive stimulation of NMDA receptors, increasing the risk of epileptic seizures. This mechanism highlights the key role of metabolites in maintaining the balance of neurotransmission and their potential part in epilepsy pathogenesis (Kristiansen et al., 2016; (Kapur and Joshi, 2021).

Mendelian Randomization (MR) is an emerging statistical method that draws on Mendel’s laws of inheritance, specifically the law of segregation and the law of independent assortment, to explore potential causal relationships between exposures and diseases (Sanderson et al., 2022). The rise and development of MR are closely linked to advancements in large-scale Genome-Wide Association Studies (GWASs) technology. GWAS has enabled the widespread identification of genetic variants associated with diseases, providing a wealth of genetic instrumental variables for MR analysis. MR uses genetic variations, particularly single-nucleotide polymorphisms (SNPs), as instrumental variables (IVs) to simulate the random allocation mechanism of randomized controlled trials (RCTs) (Smith and Ebrahim, 2003). The core advantage of MR is its ability to construct potential causal relationships between variables while avoiding the limitations of traditional observational studies where correlation does not imply causation. As genetic variations are determined at birth and do not change due to the onset of disease, the MR method can effectively avoid the problems of confounding factors and reverse causality (Davies et al., 2018). This makes MR an ideal alternative research strategy for exploring the causal relationships between exposure factors and outcomes in situations where RCTs cannot be conducted or where conducting RCTs poses ethical challenges. A recent study investigated the causal relationship between metabolism and immune cells and female reproductive diseases with excellent results through MR (Shen et al., 2024). Mendelian randomization analysis has many advantages, it also has certain limitations due to the difficulty in satisfying the horizontal pleiotropy assumption, the challenges in effectively correcting for the influence of a few genetic regulatory factors through sensitivity analysis, and the small sample sizes in most studies on metabolic quantitative trait loci.

The current understanding of the causal effect of immune cells and plasma metabolites on epilepsy is limited. This study used GWAS and a two-sample MR approach to assess the potential causal association of 731 immune cells and 1,400 plasma metabolites with the occurrence of epilepsy. Further, we investigated whether the identified candidate immune cells influence the development of epilepsy through their effects on plasma metabolites. The findings of this study not only deepen our understanding of the pathophysiological basis of epilepsy but also provide the groundwork for the development of effective epilepsy screening and prevention measures, which are significant for improving clinical outcomes.

## Methods

### Data sources

A total of 731 immune cell and 1,400 metabolite data were obtained as exposures, and their associated SNP data was sourced from the IEU GWAS (www.gwas.mrcieu.ac.uk) (accession numbers GCST0001391-GCST0002121) (Orrù et al., 2020) and the GWAS Catalog databases (www.ebi.ac.uk/gwas/) (accession numbers GCST90199621-GCST90201020) (Chen et al., 2023). Additionally, SNP data relevant to epilepsy as the outcome were derived from a large-scale genome-wide analysis encompassing 15,212 epilepsy patients and 29,677 controls (International League Against Epilepsy Consortium on Complex Epilepsies, 2018). These datasets were exclusively drawn from European populations.

### Instrumental variable selection

To select SNPs significantly associated with immune cells and metabolites as IVs for subsequent two-sample and mediation MR analyses, we employed a rigorous screening process. First, SNPs associated with the exposure factors were filtered based on a genome-wide significance threshold (*p* < 1 × 10^−5^). Second, we assessed linkage disequilibrium (LD) among SNPs using parameters of *R*^2^ < 0.001 and a clumping distance of 10,000 kb. Third, SNPs showing inconsistency in allele frequencies between exposure and outcome, as well as, palindromic and ambiguous SNPs, were excluded. Fourth, weak IVs were eliminated by calculating the F statistic using the formula $F=\frac{R^{2}\times \left(N-1-K\right)}{\left(1-R^{2}\right)\times K}$, where *R*^2^ represents the proportion of variance in the exposure explained by IVs, N is the sample size, and K is the number of IVs. SNPs with an F statistic ≥10 were considered strong IVs, while those with an F statistic <10 were considered weak IVs (Pierce et al., 2011). Finally, SNPs exhibiting associations between exposure and mediator were excluded in the MR analysis of mediation between exposure and outcome.

### Two-sample and mediation MR analysis

MR analysis was conducted using 731 immune cell and 1,400 metabolite data as exposure factors and epilepsy as the outcome, to estimate the causal relationships and mediation effects between immune cells, metabolites, and epilepsy. We adhered to the three core assumptions of MR analysis and used multiple testing methods, including inverse variance weighted (IVW), MR Egger, Wald ratio, weighted median, simple mode, and weighted mode (for detailed descriptions, refer to Supplementary method 1).

A two-step MR analysis strategy was employed. First, two-sample MR analysis (TSMR) was used to assess and select the causal effects between immune cells and epilepsy (referred to as the total effect, β0). Subsequently, reverse MR analysis was conducted to identify immune cells without causal relationships for subsequent mediation analysis. Thereafter, TSMR was employed to determine the causal relationship between metabolites and epilepsy (denoted as β2). Finally, based on the results indicating causal relationships between epilepsy and immune cells as well as metabolites, potential causal effects between them (denoted as β1) were further analyzed. The coefficient product method was utilized to calculate the mediation effects of each immune cell on epilepsy through metabolites (β1 × β2). The direct effect of immune cells on epilepsy was calculated as β0 - β1 × β2. The detailed steps were shown in the flowchart (Supplementary Figure S1).

### Sensitivity analysis

To assess if IVs exhibit heterogeneity and horizontal pleiotropy during the MR analysis, we used Cochran’s Q statistic test and MR-Egger regression intercept analysis. Heterogeneity is indicated when the *Q* value exceeds the number of IVs minus one or when *p* < 0.1. A lack of pleiotropy is inferred when the *p*-value of MR-Egger regression intercept analysis is <0.05. In the presence of pleiotropy, the corresponding SNPs were removed, and the analysis was re-conducted. Additionally, we have introduced a new sensitivity analysis. Due to the limitations of the MR-Egger test in detecting pleiotropy, a single SNP might be associated with multiple metabolites. Therefore, we excluded signals related to more than two metabolites and conducted sensitivity analysis using independent SNPs. We then performed MR analysis again to verify the robustness of the results. In addition, we conducted the leave-one-out analysis to assess the stability of the causal effects, excluding individual influential SNPs.

### Statistical analyses

Statistical analyses were conducted using the strawberry version 5.30.0.1 and R version 4.2.1 software. The following R packages were used: “TwoSampleMR” (version 0.5.6), “ieugwasr” (version 0.1.5), “variantannotation” (version 1.46.0), and “forestplot” (version 3.1.3). All the code used to generate the figures were provided in the Supplementary material.

## Results

### Instrumental variable information

We identified 18,622 and 34,844 SNPs strongly associated with 731 immune cell and 1,400 metabolite, respectively (Supplementary Tables S1, S2). Upon estimation of the effects of these SNPs on epilepsy outcomes, varying numbers of SNPs were identified to be associated with immune cells and metabolites. Among them, immune cells, such as CM DN (CD4−CD8−) AC, CD24 on IgD− CD38dim, CD28 on CD28+ DN (CD4−CD8−), CD25 on CD28+ CD4+, and CD45RA on CD39+ resting Treg, as well as, metabolites such as dihydroferulate levels, phosphate to fructose ratio, and citrate to taurocholate ratio, were each represented by a single SNP. Therefore, their MR analysis was solely conducted using the Wald ratio method. Likewise, immune cells such as CD39+ activated Treg %activated Treg, DP (CD4+CD8+) %T cell, and CD80 on monocyte, and metabolites such as N-methyl-2-pyridone-5-carboxamide levels, 3-hydroxy-2-methylpyridine sulfate levels, and succinate levels, were associated with only two SNPs; thus, their MR analysis was based solely on IVW results. The assessment of causal relationships between immune cells and metabolites revealed 269 strongly correlated SNPs (Supplementary Table S3). Of these, 10 types of immune cells exhibited causal relationships with multiple metabolites and were associated with 16–28 SNPs.

### Causal relationships between immune cells and epilepsy

**Figure 1:**
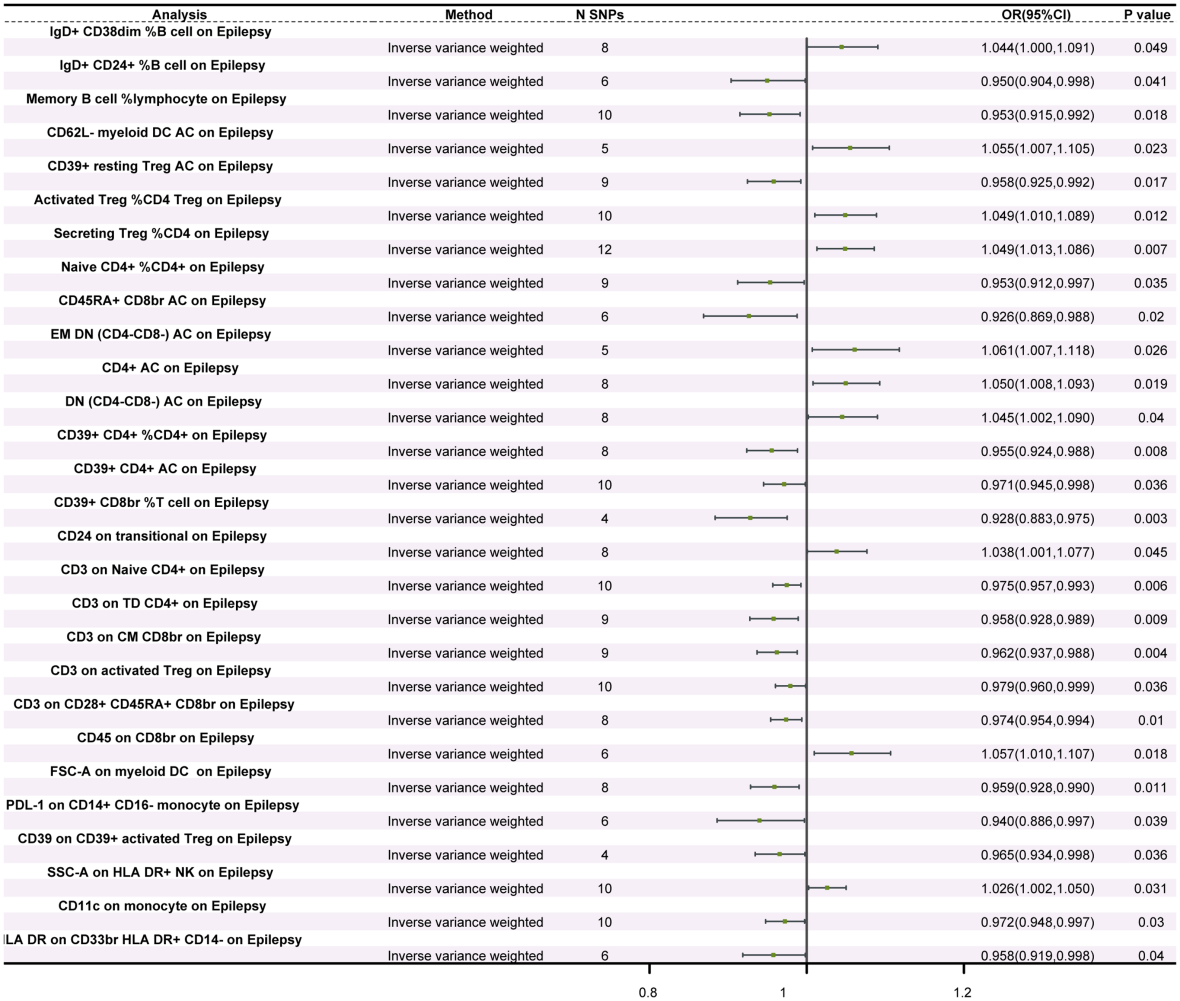
Forest plot of the causal relationship between immune cells and epilepsy. The figure illustrates the impact of different immune cells on the risk of epilepsy. Each horizontal line represents the odds ratio (OR) and its 95% confidence interval (95% CI) for a specific immune cell’s effect on epilepsy risk. The OR indicates the relationship between exposure to the immune cell and the risk of epilepsy, where OR > 1 indicates a positive correlation (increased risk) and OR < 1 indicates a negative correlation (decreased risk). A *p*-value < 0.05 is considered statistically significant.

MR analysis examining the relationship between immune cells and epilepsy revealed significant causal associations between epilepsy and 28 different subtypes of immune cells—four types of B cells, three types of classical dendritic cells (cDC), six types of maturation stages of T cells, one type of monocyte, one type of myeloid cell, four types of TBNK cells, and nine types of Treg cells. IVW results showed that 18 types of immune cells, including CD45RA+ CD8br AC (OR = 0.926, 95%CI: 0.869–0.988, *p* = 0.020), CD39+ CD8br %T cell (OR = 0.928, 95%CI: 0.883–0.975, *p* = 0.003), PDL-1 on CD14+ CD16− monocyte (OR = 0.940, 95%CI: 0.886–0.997, *p* = 0.039), and IgD+ CD24+ %B cell (OR = 0.950, 95%CI: 0.904–0.998, *p* = 0.041), had a protective effect on epilepsy occurrence. Furthermore, 10 types of immune cells, including SSC-A on HLA DR+ NK (OR = 1.026, 95%CI: 1.002–1.050, *p* = 0.031), CD24 on transitional (OR = 1.038, 95%CI: 1.000–1.077, *p* = 0.045), and IgD+ CD38dim %B cell (OR = 1.044, 95%CI: 1.000–1.091, *p* = 0.049), were identified as risk factors for epilepsy occurrence (Figure 1; Supplementary Figure S2; Supplementary Table S4). Sensitivity analysis results indicated that both Cochran’s Q tests for MR-Egger and IVW methods showed no heterogeneity (*p* > 0.05) among the 28 immune cell types (refer to Supplementary Table S5). MR-Egger regression intercept analysis also did not detect the presence of horizontal pleiotropy (p > 0.05) (Supplementary Table S6). Moreover, the leave-one-out sensitivity analysis revealed that the causal relationships between the 28 immune cells and epilepsy were not driven by a single SNP (Supplementary Figure S3). Additionally, reverse MR analysis found no causal relationship between epilepsy and the 28 immune cells (Supplementary Table S7).

### Causal relationships between metabolites and epilepsy

**Figure 2:**
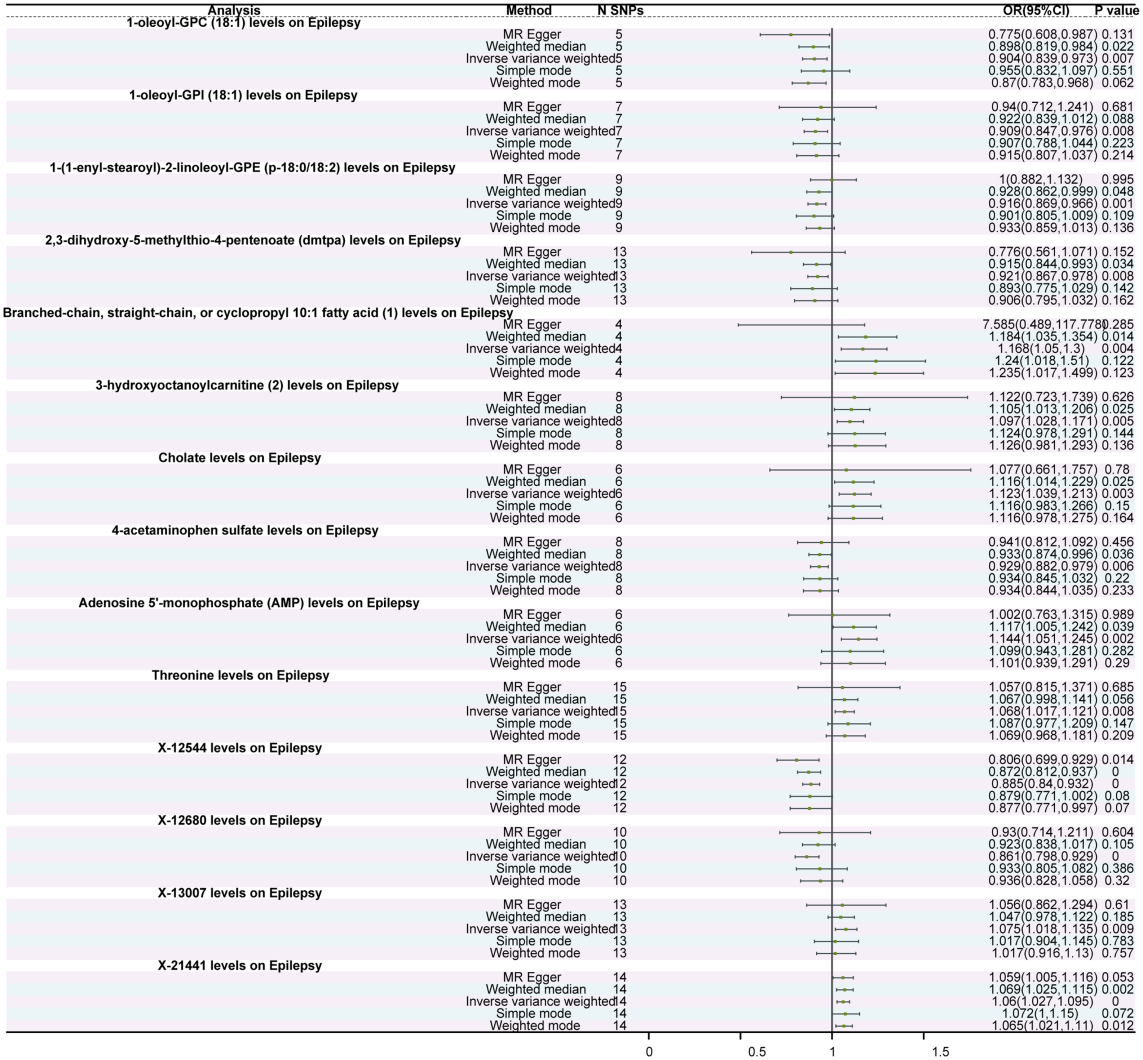
Forest plot of the causal relationship between metabolites and epilepsy. The figure depicts the impact of different immune cells on the risk of epilepsy. Each horizontal line represents the odds ratio (OR) and its 95% confidence interval (95% CI) for a specific immune cell’s effect on epilepsy risk. The OR indicates the relationship between exposure to the immune cell and the risk of epilepsy, where OR > 1 indicates a positive correlation (increased risk) and OR < 1 indicates a negative correlation (decreased risk). A *p*-value < 0.05 is considered statistically significant.

**Figure 3:**
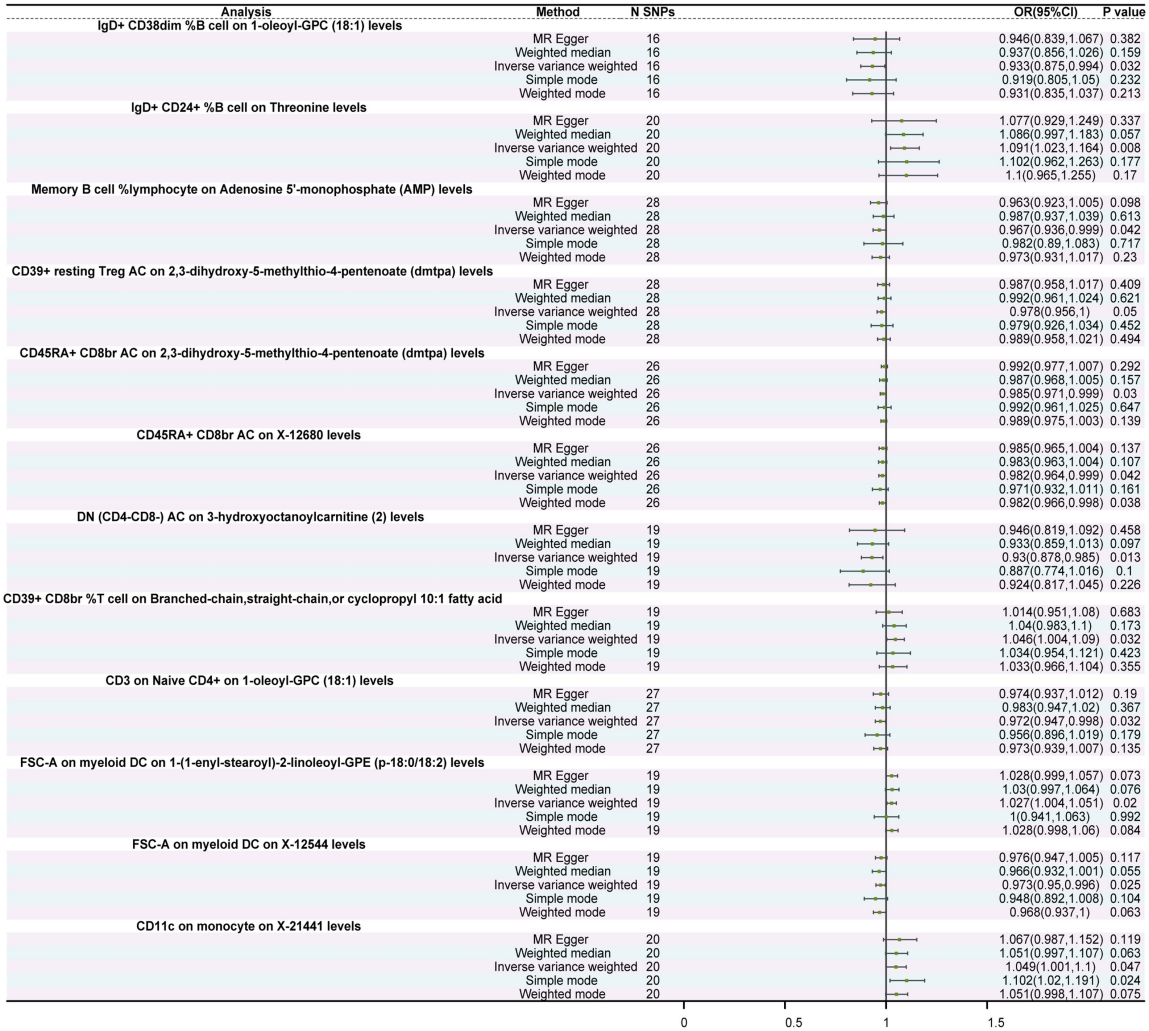
Forest plot of the causal relationship between immune cells and metabolites. The figure illustrates the causal relationship between different immune cells and metabolites. Each horizontal line represents the odds ratio (OR) and its 95% confidence interval (95% CI) for the effect of a specific immune cell on metabolites. The OR indicates the relationship between the immune cell and metabolites, where OR > 1 indicates a positive correlation (increased effect), and OR < 1 indicates a negative correlation (decreased effect). A *p*-value < 0.05 is considered statistically significant.

Among the 1,400 metabolites assessed, IVW analysis revealed 14 metabolites with significant causal relationships with epilepsy. Of these, seven were protective factors and seven were risk factors (Figure 2). Protective factors included 1-oleoyl-GPC (18:1) levels (OR = 0.904, 95%CI: 0.839–0.973, *p* = 0.007), 2,3-dihydroxy-5-methylthio-4-pentenoate (DMTPA) levels (OR = 0.921, 95%CI: 0.867–0.978, *p* = 0.008), 1-oleoyl-GPI (18,1) levels (OR = 0.909, 95%CI: 0.847–0.976, p = 0.008), and 1-(1-enyl-stearoyl)-2-linoleoyl-GPE (p-18:0/18:2) levels (OR = 0.916, 95%CI: 0.869–0.966, *p* = 0.001). Risk factors included cholate levels (OR = 1.123, 95%CI: 1.039–1.213, *p* = 0.003), Threonine levels (OR = 1.068, 95%CI: 1.017–1.121, p = 0.008), and adenosine 5′-monophosphate (AMP) levels (OR = 1.144, 95%CI: 1.051–1.245, *p* = 0.002) (Figure 3; Supplementary Figure S4; Supplementary Table S8). Furthermore, except for threonine X-12680 levels, the weighted median method supported the causal relationships between the remaining 14 metabolites and epilepsy. In the sensitivity analysis, neither Cochran’s Q tests nor MR-Egger regression intercept analysis detected heterogeneity or pleiotropy (Supplementary Tables S9, S10). Additionally, we excluded 2,957 SNPs associated with more than two metabolites (Supplementary Table S11), leaving 26,345 independent SNPs for the sensitivity analysis (Supplementary Table S12). The results showed that 18 metabolites were associated with epilepsy, and the findings remained robust for all except for 5-acetylamino-6-formylamino-3-methyluracil levels, N-acetyl phenylalanine levels, X-24978 levels, and Citramalate levels (Supplementary Table S13). Moreover, the leave-one-out analysis showed that these results were relatively robust, with minimal influence from individual SNPs (Supplementary Figure S5).

### Causal relationships between immune cells and metabolites

MR analysis revealed significant causal relationships between 10 types of immune cells and metabolites. Notably, IgD+ CD24+ %B cells exhibited a significant promoting effect on threonine levels (OR = 1.091, 95%CI: 1.023–1.164, *p* = 0.008). Additionally, memory B cell % lymphocytes exerted a negative regulatory effect on Adenosine 5′-monophosphate (AMP) levels (OR = 0.967, 95%CI: 0.936–0.999, *p* = 0.042). Furthermore, DN (CD4−CD8−) AC showed an inverse causal relationship with 3-hydroxyoctanoylcarnitine (2) levels (OR = 0.930, 95%CI: 0.878–0.985, *p* = 0.013) in that for each standard deviation (SD) increase in DN (CD4−CD8−) AC, 3-hydroxyoctanoylcarnitine (2) levels decreased by 0.07 times (Figure 3; Supplementary Table S12). Moreover, both Cochran’s Q test and MR-Egger regression intercept analysis showed no statistical significance between immune cells and metabolites (Supplementary Tables S13, S14), indicating the absence of heterogeneity and pleiotropy in these IVs, thus enhancing the reliability of these results.

### Metabolites mediating the causal relationship between immune cells and epilepsy

Further analysis revealed that eight metabolites mediated the effects of six immune cell types on epilepsy (Table 1). Of these, 3-hydroxyoctanoylcarnitine (2) levels exhibited the highest mediating effect, mediating 15.3% (95%CI, −0.008, −30.6%, *p* = 0.049) of the effect of DN (CD4−CD8−) AC on epilepsy. AMP levels mediated 9.34% (95%CI, 0.050, 18.6%, p = 0.049) of the effect of memory B cell % lymphocytes on epilepsy. Additionally, DMTPA (95%CI, −3.15, −0.135%, *p* = 0.033) and X-12680 levels (95%CI, −7.12, −0.098%, *p* = 0.044) mediated 1.64 and 3.61%, respectively, of the total effect of CD45RA+ CD8br AC on epilepsy. 1-(1-enyl-stearoyl)-2-linoleoyl-GPE (p-18:0/18:2) levels (95%CI, 0.668, 10.6%, *p* = 0.026) and X-12544 levels (95%CI, −15.1, −0.856%, *p* = 0.028) contributed 5.63 and 8%, respectively, on the causal effect of FSC-A on myeloid DC on epilepsy. The schematic diagram of the relationship between immune cells, metabolites, their intermediary connections, and epilepsy was shown in Supplementary Figure S6.

**Table 1:** Mediation effects of metabolites on the relationship between immune cells and epilepsy.

Immune cell	Metabolite	Outcome	Mediated proportion	*p* value
Memory B cell %lymphocyte	Adenosine 5′-monophosphate (AMP) levels	Epilepsy	9.34% (0.050, 18.6%)	0.049
CD45RA+ CD8br AC	2,3-dihydroxy-5-methylthio-4-pentenoate (dmtpa) levels	Epilepsy	−1.64% (−3.15, −0.135%)	0.033
CD45RA+ CD8br AC	X-12680 levels	Epilepsy	−3.61% (−7.12, −0.098%)	0.044
DN (CD4−CD8−) AC	3-hydroxyoctanoylcarnitine (2) levels	Epilepsy	−15.3% (−0.008, −30.6%)	0.049
CD39+ CD8br %T cell	Branched-chain, straight-chain, or cyclopropyl 10:1 fatty acid (1) levels	Epilepsy	−9.43% (−18.4, −0.467%)	0.039
CD3 on Naive CD4+	1-oleoyl-GPC (18:1) levels	Epilepsy	−11.2% (−22, −0.535%)	0.040
FSC-A on myeloid DC	1-(1-enyl-stearoyl)-2-linoleoyl-GPE (p-18:0/18:2) levels	Epilepsy	5.63% (0.668, 10.6%)	0.026
FSC-A on myeloid DC	X-12544 levels	Epilepsy	−8% (−15.1, −0.856%)	0.028

## Discussion

An increasing number of studies are exploring the genetic, immunologic, and metabolic bases of epilepsy, given by fast-growing prevalence (Ravizza et al., 2008; Vezzani et al., 2011; Auvin, 2012; Bejarano and Rodríguez-Navarro, 2015; Almannai et al., 2021). Advances in immunology and metabolomics have provided valuable insights into understanding the pathogenesis of epilepsy. In contrast to these observational studies, we employed MR using genetic variation as instrumental variables and summary statistics from GWAS to reveal causal relationships between epilepsy risk and levels of immune cells and metabolites.

Our findings revealed that 28 immune phenotypes were causally associated with epilepsy. Of these immune phenotypes, 18 were correlated with a reduced risk of epilepsy, whereas 10 were associated with an increased risk of epilepsy. Additionally, we found that among the 1,400 metabolites investigated, 14 were causally linked to the occurrence of epilepsy—seven were positively correlated and seven were negatively correlated with epilepsy occurrence. Furthermore, eight metabolites mediated the effects of six immune cells on epilepsy. In particular, IgD+ CD38dim %B cell on 1-oleoyl-GPC (18:1) levels, memory B cell %lymphocyte on AMP levels, CD39+ resting Treg AC on DMTPA levels, CD45RA+ CD8br AC on DMTPA levels, CD45RA+ CD8br AC on X-12680 levels, DN (CD4−CD8−) AC on 3-hydroxyoctanoylcarnitine (2) levels, CD3 on Naive CD4+ on 1-oleoyl-GPC (18,1) levels, and FSC-A on myeloid DC on X-12544 levels were associated with reduced epilepsy risk. Whereas, IgD+ CD24+ %B cell on threonine levels, CD39+ CD8br %T cell on branched-chain, straight-chain, or cyclopropyl 10:1 fatty acids, FSC-A and myeloid DC on 1-(1-enyl-stearoyl)-2-linoleoyl-GPE (p-18:0/18:2) levels, and CD11c on monocyte on X-21441 levels were associated with increased epilepsy risk.

Our results indicate that CD3 and CD39 may play important roles in various types of T cells. Inflammation mediated by T cells is considered a key factor in the occurrence of epilepsy (Vezzani et al., 2011). The activation of CD3 promotes the differentiation and proliferation of T cells, releasing various inflammatory factors, such as interferon-gamma (IFN-γ) and tumor necrosis factor-alpha (TNF-α). These inflammatory factors can enhance the activity of immune cells in the brain and promote the production of inflammatory mediators, further affecting the excitability of neurons, leading to the occurrence and progression of epilepsy (Smith-Garvin et al., 2009). In addition, the regulatory role of CD3 may also affect the subset distribution and function of T cells, such as the dysfunction of regulatory T cells (Treg), thereby affecting the immune system’s response to the brain (Riazi et al., 2010). CD39 is a cell surface enzyme highly expressed in astrocytes and endothelial cells; its main function is to catalyze the hydrolysis of extracellular ATP and ADP, generating AMP (Braun et al., 2000). Extracellular ATP, as an inflammatory mediator, can activate immune cells and promote the development of inflammatory reactions. Moreover, a previous study has shown that targeting purinergic P2 receptors as pharmacological targets can effectively regulate the occurrence of epilepsy, the inflammatory process, and brain damage caused by epileptic seizures (Engel et al., 2016).

The impact of AMP on the occurrence of epilepsy is multifaceted. First, AMP can be metabolized into adenosine, which acts through G protein-coupled adenosine receptors to participate in the onset of epilepsy (Boison and Jarvis, 2021). Additionally, the accumulation of AMP within cells can lead to the activation of AMP-activated protein kinase (AMPK), which enhances the cell’s energy utilization efficiency (Liu et al., 2020), however, excessive activation may lead to cellular dysfunction, thereby triggering epileptic seizures. AMP may also be involved in the pathogenesis of epilepsy through other mechanisms, such as affecting the function of ion channels or regulating the release of neuroexcitatory agents (Benarroch, 2013). DMTPA is an amino acid metabolite derived from S-adenosylmethionine, significantly associated with acute kidney injury and hypertension (Bajaj et al., 2021; Wang et al., 2021). It may promote the occurrence of epilepsy by affecting cerebral hemodynamic changes such as ischemia, microangiopathy, and cerebral arteriosclerosis (Gasparini et al., 2019). Yang et al. indicated that X-12680 is associated with Alzheimer’s disease (AD) risk and has a role in reducing this risk (Yang et al., 2024). In our study, we found similar results. Both AD and epilepsy are related to abnormal nervous system function. We hypothesize that X-12680 levels may be beneficial for neuronal repair, thereby protecting nervous system function.

Metabolite research has been conducted for neurological diseases. Darst et al. revealed that 7 out of 1,097 metabolites are related to the executive function trajectory of AD (Darst et al., 2021). A recent study suggests a significant bidirectional causal relationship between generalized convulsive epilepsy and inflammatory factors (Wang et al., 2023), suggesting the potential mechanisms by which inflammatory metabolites influence the occurrence of epilepsy.

In this study, we used new metabolite datasets to explore their impact on epilepsy risk and found a causal relationship between 14 metabolites and the occurrence of epilepsy. These findings highlight the significant role that metabolites play in the development of epilepsy. Of these, threonine metabolism plays a crucial role in the epileptic mechanism in humans, rats, and mice (Lai et al., 2022). Threonine participates in the synthesis of neurotransmitters such as glycine and serine, triggers excitatory neurotransmission, and thereby influences the excitatory and inhibitory balance of neurons, promoting the onset and development of epilepsy (Hernandes and Troncone, 2009).

Additionally, in our study, we found that six types of immune cells mediate the regulation of eight metabolites, influencing the occurrence of epilepsy. The mechanism by which immune cells regulate metabolites to influence the occurrence of epilepsy is not yet clear. We hypothesize that this mediating effect may be related to energy metabolism, neurotransmitter transmission, and inflammatory responses (Vezzani and Friedman, 2011; Marchi et al., 2014).

Our study’s novelty is first reflected in our use of diverse immune cell characteristics and metabolites as research subjects, revealing their potential causal relationship with the occurrence of epilepsy. Second, by employing mediation analysis, we provide a theoretical basis for understanding the mechanism by which immune cells influence the development of epilepsy through the regulation of metabolites (refer to Supplementary Figure S6). This offers valuable references and insights for future research.

Our study still has some limitations. First, the GWAS data we used were all from European cohorts. Given the genetic diversity between different races, the validity of our findings may not apply to other ethnic populations. Second, the sample size of the immune cell and metabolite data used in this study is relatively small, which may affect the reliability and generalizability of the results. Therefore, in the future, the results must be validated in a larger sample to improve the statistical power of the study and ensure the robustness and credibility of the conclusions. Finally, our study focused on the general population of patients with epilepsy without distinguishing between different subtypes of epilepsy, limiting the exploration of personalized treatment strategies for individuals with epilepsy.

## Conclusion

Our study revealed the critical roles of immune cells and metabolites in the development of epilepsy. It has identified potential targets for future interventions, such as modulating levels of CD3, CD39, or threonine, which could become new strategies for preventing and treating epilepsy. Future research should include functional studies to further explore the roles and levels of immune cells and metabolites in cellular and animal models. These studies will deepen our understanding of the specific pathways and interactions involving immune and metabolic features in the pathogenesis of epilepsy, thereby validating our findings and enhancing their clinical translational potential.

## Data availability statement

The original contributions presented in the study are included in the article/Supplementary material, further inquiries can be directed to the corresponding author.

## Author contributions

KW: Writing – original draft, Software, Investigation, Formal analysis. JY: Writing – original draft, Methodology, Data curation. WX: Writing – review & editing, Supervision, Investigation. LW: Writing – review & editing, Visualization, Methodology. YW: Writing – review & editing, Supervision, Methodology.
